# Fast Acceleration of 2D Wave Propagation Simulations Using Modern Computational Accelerators

**DOI:** 10.1371/journal.pone.0086484

**Published:** 2014-01-30

**Authors:** Wei Wang, Lifan Xu, John Cavazos, Howie H. Huang, Matthew Kay

**Affiliations:** 1 Computer and Information Sciences Department, University of Delaware, Newark, Delaware, United States of America; 2 Electrical and Computer Engineering Department, George Washington University, Washington, DC, United States of America; University of Warwick, United Kingdom

## Abstract

Recent developments in modern computational accelerators like Graphics Processing Units (GPUs) and coprocessors provide great opportunities for making scientific applications run faster than ever before. However, efficient parallelization of scientific code using new programming tools like CUDA requires a high level of expertise that is not available to many scientists. This, plus the fact that parallelized code is usually not portable to different architectures, creates major challenges for exploiting the full capabilities of modern computational accelerators. In this work, we sought to overcome these challenges by studying how to achieve both automated parallelization using OpenACC and enhanced portability using OpenCL. We applied our parallelization schemes using GPUs as well as Intel Many Integrated Core (MIC) coprocessor to reduce the run time of wave propagation simulations. We used a well-established 2D cardiac action potential model as a specific case-study. To the best of our knowledge, we are the first to study auto-parallelization of 2D cardiac wave propagation simulations using OpenACC. Our results identify several approaches that provide substantial speedups. The OpenACC-generated GPU code achieved more than 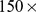 speedup above the sequential implementation and required the addition of only a few OpenACC pragmas to the code. An OpenCL implementation provided speedups on GPUs of at least 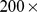 faster than the sequential implementation and 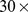 faster than a parallelized OpenMP implementation. An implementation of OpenMP on Intel MIC coprocessor provided speedups of 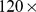 with only a few code changes to the sequential implementation. We highlight that OpenACC provides an automatic, efficient, and portable approach to achieve parallelization of 2D cardiac wave simulations on GPUs. Our approach of using OpenACC, OpenCL, and OpenMP to parallelize this particular model on modern computational accelerators should be applicable to other computational models of wave propagation in multi-dimensional media.

## Introduction

Recent developments in the field of high performance computing have greatly expanded the computational capabilities and application of Graphics Processing Units (GPUs). Using GPUs to perform computations that are typically handled by a CPU is known as General Purpose computation on GPUs (GPGPU). To name a few, GPUs are now used in the fields of bioinformatics [1], signal processing [2], astronomy [3], weather forecasting [4], and molecular modeling [5]. In addition to GPUs, Intel's new Many Integrated Core (MIC) architecture also provides a powerful parallel platform for complex computations. The Intel Xeon Phi is the first accelerator based on the MIC architecture and is expected to accelerate oil exploration, climate simulation, and financial analyses, as well as other applications [6]. While new accelerators promise improved computational performance, the use of software tools (like CUDA programming language) to drive parallelism of the accelerators requires expertise that is not widely available. In addition, CUDA code will only run on NVIDIA GPUs, thereby limiting its portability to other accelerators.

In this work, we sought to overcome the challenges of CUDA by studying how to achieve both automated parallelization using OpenACC (a directive based language extension similar to OpenMP) and enhanced portability using OpenCL. We applied our parallelization schemes using GPUs as well as Intel MIC-architecture accelerator to reduce the run time of wave propagation simulations. We used a well-established 2D cardiac action potential model as a specific case-study.

Models of cardiac wave propagation often require a large number of computations at each model node at each time step to compute the value of numerous ionic currents at the node. A computational approach that uses parallel processing on GPUs or millions of cores provides a huge performance increase over any sequential approach. For example, Neic et al accelerated cardiac biodomain propagation simulations using a cluster of GPUs which achieved up to 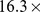 speedups over parallelized CPU implementations [7]. Mirin et al simulated thousands of heartbeats at a resolution of 0.1 mm using more than one million cores [8]. They were also able to simulate human heart function over 1200 times faster compared with any published results in the field [9]. Unlike prior work that focused on either using one programming language for parallelization on GPUs or multiple programming models targeting CPUs [7]–[12], we studied multiple parallelization approaches for running 2D cardiac wave propagation simulations.

We first used OpenACC to automatically generate CUDA and OpenCL GPU code that runs on NVIDIA GPUs. In addition, we developed CUDA and OpenCL implementations for parallelization on NVIDIA GPUs, as well as an OpenMP implementation of the same model for parallelization on Intel CPUs. In this way we could compare the performance of different parallel computing techniques. By altering the number of threads in the OpenMP implementation, we were able to achieve good speedups on Intel Xeon Phi accelerator.

The contributions of this paper are two fold: 1) We parallelized a 2D cardiac wave propagation model using both manual and automatic parallelization paradigms including CUDA, OpenCL, and OpenACC. In particular, auto-parallelizing our model using OpenACC is an excellent example of achieving parallelism in an efficient and effective way. 2) We applied OpenCL, OpenACC, and OpenMP to the problem of simulating cardiac wave propagation so that parallelism from different architectures could be explored. We found that this approach provided excellent speedups of the model on GPUs and the Intel MIC-architecture accelerator. Our results indicate that this is a very useful approach for solving computational models of wave propagation in multi-dimensional media using newly-available computational accelerators.

## Methods

### High Performance Computing on GPUs

GPUs are massively parallel multi-threaded devices capable of executing a large number of active threads concurrently. A GPU consists of multiple streaming multiprocessors, each of which contains multiple scalar processor cores. For example, NVIDIA's Fermi architecture GPU card Tesla C2050 contains 14 such multiprocessors, each of which contains 32 cores, for a total of 448 cores. In addition, the GPU has several types of memory, most notably the main device memory (global memory) and the on-chip memory shared between all cores of a single multiprocessor (shared memory).

GPUs achieve high-performance computing through the massively parallel processing power of hundreds or even thousands of compute cores. There are two popular programming schemes for GPUs. The first is CUDA (Compute Unified Device Architecture) [13], a parallel programming model that delivers the high performance of NVIDIA's graphics processor technology to general purpose GPU computing. Applications written using CUDA can run on a wide variety of NVIDIA GPUs. The second is OpenCL (Open Computing Language) [14], a framework similar to CUDA. Applications written in OpenCL can be executed across heterogeneous platforms. Specifically, OpenCL applications can run on AMD GPUs, NVIDIA GPUs, AMD CPUs, Intel CPUs, and Intel coprocessors. Recent development of directive-based GPU programming allows the programmer to target GPU by simply placing pragmas in sequential code with the compiler generating either CUDA or OpenCL code to realize parallelism [15]. OpenACC [16] is such a directive-based GPU programming tool that helps drive GPU parallelism.

### Many Integrated Core Architecture

Intel MIC-architecture accelerator card is the most up-to-date product from Intel. A MIC card usually contains 60 or 61 cores, each core supports 4 hardware threads. One notable feature is that it has 512-bit wide SIMD vectors which provides fine granularity vectorization parallelism. A single instruction can operate on 8 adjacent double-precision floating point data or 16 single-precision floating point data. Intel MIC accelerator achieves high-performance computing through the hardware threads and the wide vector registers. Comparing to GPUs, a whole application can execute on the MIC accelerator. The programming languages for MIC accelerator include OpenMP, OpenCL, and MPI. In this work, we use OpenMP and OpenCL to exploit the parallelism of one MIC card.

### Cardiac Wave Propagation Model

Our goal was to study the computational speedups for simulating cardiac electrical wave propagation that are provided by multiple hardware platforms and several programming schemes. We chose to work with a relatively straight-forward 2D implementation of a well-known cardiac action potential model (Beeler-Reuter). This model simplified porting the code between hardware platforms and programming schemes. Although the model is not as complex as models used in state-of-the-art simulations [7], [10], we used it as a case-study that could provide a simplified, systematic approach for comparing modern parallel programming tools. Our experience in parallelizing this model will provide insights for others seeking to parallelize similar cardiac models [17] and other propagation models, such as convection and diffusion models [18], [19], seismic wave propagation models [20], and tumor growth and drug transport models [21].

**Figure 1:**
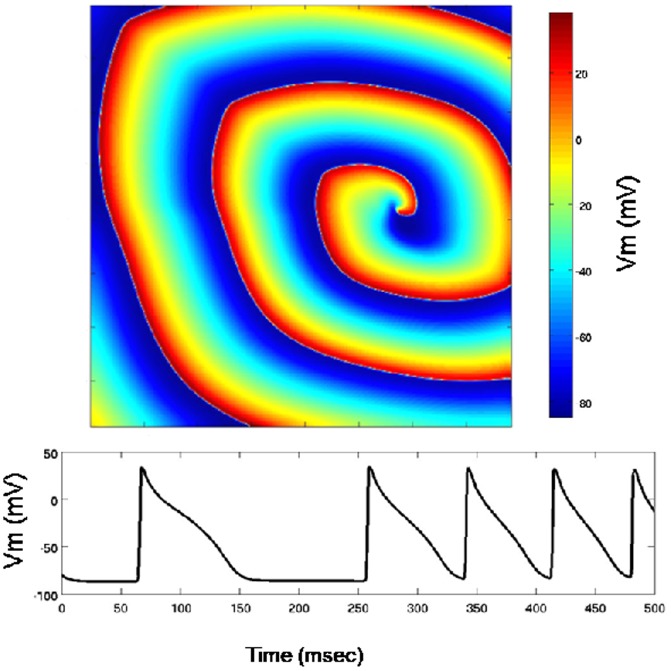
Simulation of a single rotor. Top: an image showing cardiac electrical wave propagation as spatial fluctuations of transmembrane potential. A single rotating wave (rotor) is shown. Bottom: action potentials at one node are shown as the temporal variation of transmembrane potential at the node.

**Figure 2:**
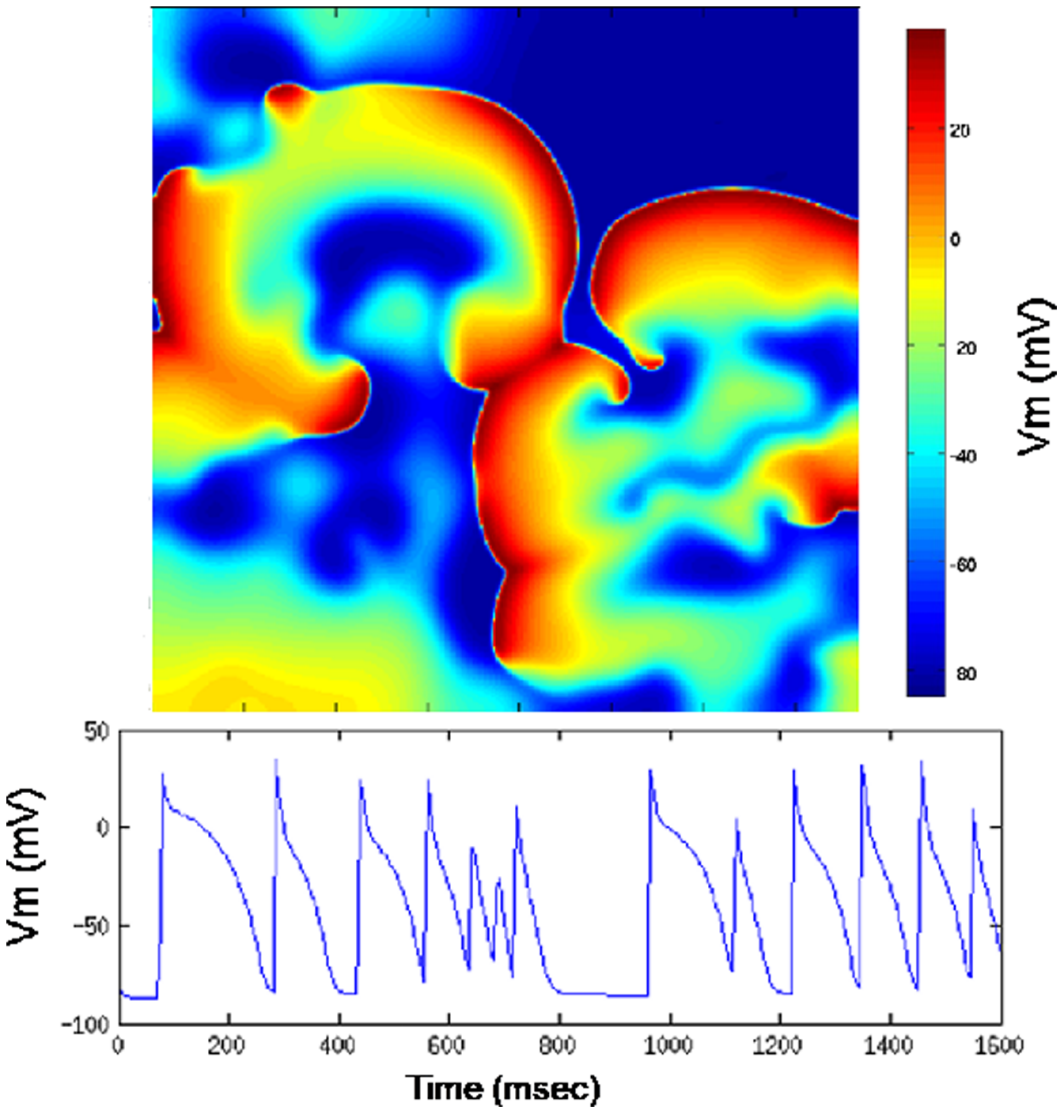
Simulation of rotor breakup and fibrillatory activity. Top: an image of transmembrane potential showing complex wave activity. Bottom: action potentials at one node are shown as the temporal variation of transmembrane potential at the node.

In our cardiac wave model, cardiac tissue is modeled as a large geometrical network of nodes that are electrically coupled. The electrical potential of the cardiac cell membrane at each node is represented as a set of partial differential equations (shown in Equation 1).
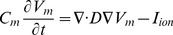
(1)Transmembrane potential (Vm) at each node in a rectilinear 2D grid (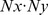) was computed using a continuum approach with no-flux boundary conditions and finite difference integration [22], [23]. Standard Euler time-stepping was used. Cardiac membrane ionic current kinetics (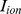, 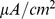) were computed using the Drouhard-Roberge formulation of the inward sodium current (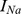) [24] and the Beeler-Reuter formulations of the slow inward current (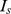), time independent potassium current (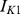), and time-activated outward current (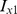) [25]. These currents are represented as complex ordinary differential equations. We simulated functional wave reentry (one rotor), and rotor wave breakup with subsequent fibrillatory wave activity. Figure 1 shows an example of simulating reentrant activity with one rotor and Figure 2 shows rotor breakup and fibrillatory activity. For wave reentry, the fiber orientation was typically set at 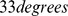; diffusion coefficient along fibers was 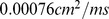 and diffusion across fibers was 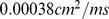. For rotor breakup and fibrillatory wave activity, the fiber orientation was set to 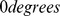; diffusion coefficient along fibers and across fibers were both 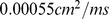. All simulations were checked for accuracy and numerical stability.

**Figure 3:**
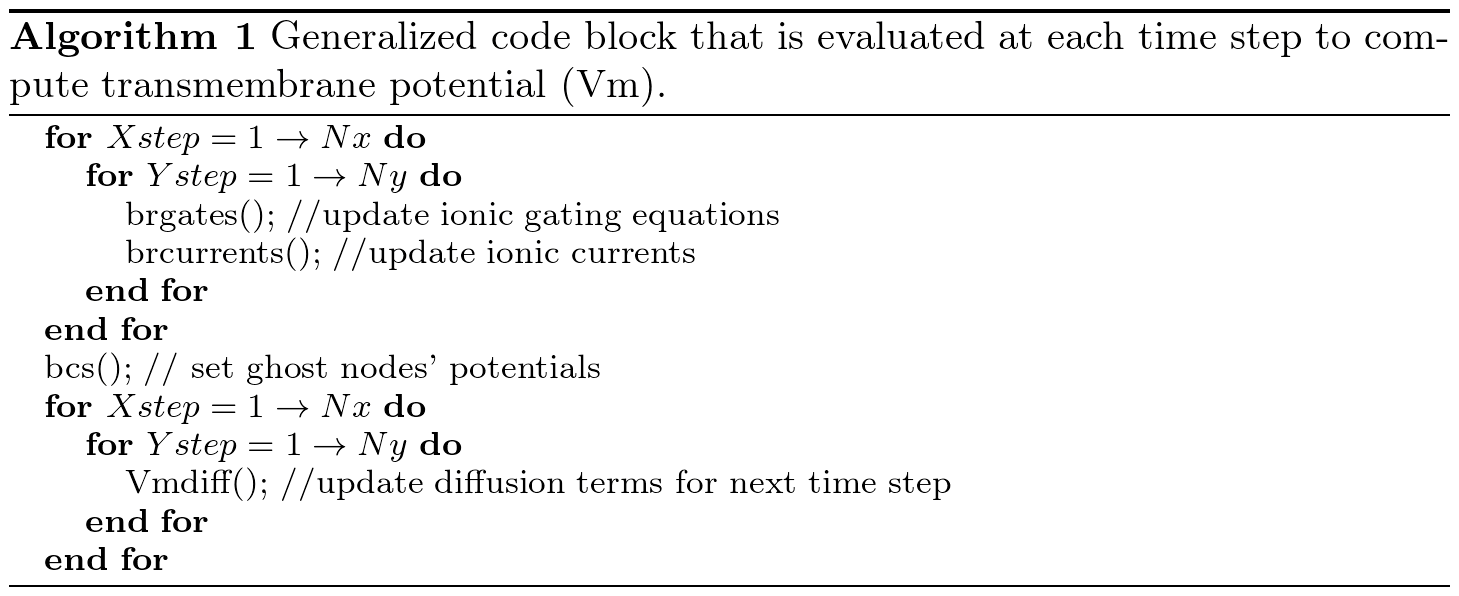
The original algorithm.

The general approach for solving the model is shown in Figure 3. The differential equations were evaluated independently at each grid node at each time step. Therefore, within each time step there is no data inter-dependency, which fits the modern computational architectures quite well. There is potential for programming tools that exploit data-level parallelism to provide dramatic computational speedups.

### GPU Implementations

As shown in Figure 3, the general algorithm loops through each node in a 2D grid. Xstep is the coordinate of the X direction and Ystep is the coordinate of the Y direction. Inside the loop, the same set of functions is evaluated at each node. The temporal loop is outside the nested spatial loops. Because of the sequential structure of the program, total computational time is proportional to the domain area, which means that large spatial domains require significantly longer computational times.

Parallel implementations of N by N dimensional cardiac models are relatively straightforward because once diffusion currents have been computed there is no data dependency between neighboring nodes for a particular timestep. Because of this, the differential equations that represent myocyte electrophysiology (the brgates() and brcurrents() functions) can be evaluated at each node, in almost any sequence.

### CUDA and OpenCL Implementations

We parallelized the cardiac model using CUDA and OpenCL. In CUDA and OpenCL, multiple threads execute the same instructions but the data processed by these threads might be on different nodes. Take CUDA for example [13], a GPU device is conceptualized as a grid containing a large number of equally-shaped blocks, into which the threads are grouped. The parameters of dimGrid and dimBlock define how the blocks (threads) align in the grid (block). Each thread block in the grid executes on a multiprocessor and threads in the block execute on multiple cores inside the multiprocessor. The simulation input set is a 2D grid containing 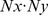 nodes (Nx columns and Ny rows of nodes). We mapped one GPU thread to one node. To achieve maximum parallelism, the optimal setting for block size must be identified. As mentioned before, inside each GPU there are many multiprocessors and each contains multiple cores. When the GPU code is executing on a GPU, all of the blocks are evenly assigned to the multiprocessors. If a multiprocessor contains 32 cores and a block has 64 threads, the first 32 threads will be executed on these 32 cores first then the rest of the threads will be swept in. Assigning large number of threads in one block may increase the occupancy of a multiprocessor, thereby keeping all cores busy. On the other hand, due to the limited resources like registers, shared memory, etc. that are available in a GPU, a larger block may increase the pressure on these resources. To find the best block size for our model, we tested our code using various block sizes (i.e. different BlockX and BlockY values) and node numbers (i.e. different Nx and Ny values) on different GPUs. We found that the sweet spot for our cardiac model is 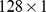 for both CUDA and OpenCL implementations running on both GPU cards.

The CUDA implementation of our cardiac model is computationally efficient and provides substantial speedups compared to a sequential CPU implementation. However, CUDA code can only run on supported NVIDIA GPUs. To address this limitation we developed the OpenCL version of the model to support GPU parallelization across platforms. In this work, the OpenCL implementation was tested on Intel MIC accelerator card in addition to NVIDIA GPUs. The general architecture of our OpenCL implementation is the same as the CUDA implementation. One thread was used to solve the equations at one node in the model.

### OpenACC Implementation

**Figure 4:**
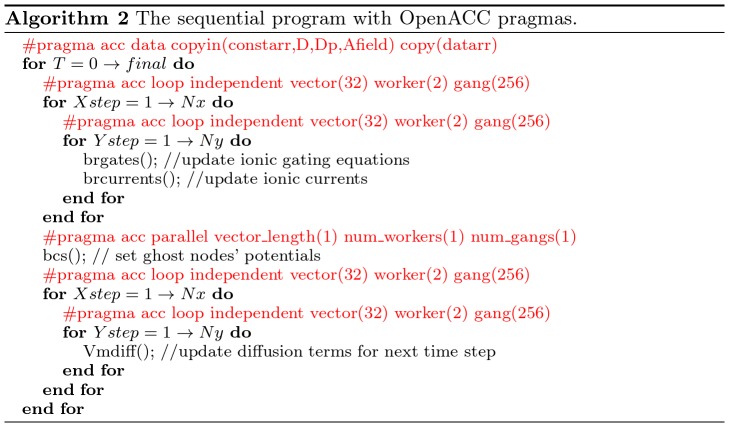
The OpenACC version of the algorithm.

Since we have identified the hotspot of the sequential program, we can add OpenACC directives to offload the code block to run on accelerators like GPUs and coprocessors. Figure 4 shows the code from Figure 3 with OpenACC pragmas.

In the code block with OpenACC pragmas, “#pragma acc data” specify which data should be copied to the accelerator (using “copyin”), to/from the accelerator (using “copy”). The “#pragma acc loop” specifies the loop that would be parallelized by OpenACC. The “vector”, “worker”, and “gang” specifies the thread configurations similar to block size and grid size in OpenCL/CUDA. For example, Algorithm 2 shown in Figure 4 is configured to have a grid size of 256 (gang) and a block size of 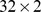 (vector,worker). Note bcs function needs to be executed in serialized manner, thus the thread configuration specifies the vector length, the number of workers, and the number of gangs to be 1. The pragmas shown in Algorithm 2 are about all that were needed to drive the generation of efficient parallel code.

### OpenMP Implementation

We parallelized the CPU code using OpenMP [26] to provide more perspectives in showing the speedups of parallel GPU implementations. We have two versions of the OpenMP implementation with different number of threads configured for CPUs and MIC accelerator respectively. Using OpenMP directive, we can specify as many threads as the number of cores in a single machine so that the best speedup results would be achieved (on this single machine). For the OpenMP implementation running on CPU, we only used one machine that contained 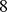 cores. In this case we created 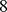 CPU threads and these threads divide among themselves all the computations. With these multiple threads, one of the spatial loops shown in Figure 3 could be parallelized. In the case of MIC card, we created as many threads as the number of hardware threads available. In exploiting the vectorization power of MIC card, we performed loop unswitching optimization to the OpenMP code. The optimization was used because the array accesses involved 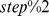 in every statement of the computation region. Loop unswitching could yield better vectorized code in this case.

## Results

In this section, we report the results of experiments with our CUDA, OpenCL, and OpenACC GPU implementations. We also report the results of comparing performances of the GPU implementations with OpenMP implementation. In the end of this section, we report the speedups from Intel MIC architecture accelerator.

### CUDA and OpenCL Implementations

#### Hardware

Our GPU implementations of the model were tested on two different GPU cards: Fermi-architecture GPU card Tesla C2050 and Kepler-architecture GPU card Tesla K20. In the following sections, we refer to them as Fermi GPU and Kepler GPU respectively. CPU-based implementations were also tested on the machines that hosted each GPU card. The machines hosting the Fermi GPU had 2 Intel E5530 2.4 GHz Quad Core Nehalem processors (8 cores total) and 24 GB memory. The machine hosting the Tesla K20 GPU card had 2 AMD Opteron 6320 2.8 GHz Eight Core processors (16 cores total) and 16 GB memory.

The Fermi GPU had 14 multiprocessors, each with 32 cores (448 cores total) clocked at 1.15 GHz and 3 GB global memory. The Kepler GPU card had 13 multiprocessors, each multiprocessor containing 192 cores (2496 cores total) clocked at 706 MHz. It had 4800 MB global memory. The peak double precision floating point performance for Fermi GPU and and Kepler GPU was 515GFlops and 1.17TFlops respectively. These hardware configurations were also used for the experiments comparing with OpenMP implementations.

#### Scalability and Performance

The computational performance of the GPU implementations (running with the best block size configuration 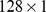) was studied by increasing the grid size and measuring total run time on each hardware platform. Simulations of reentrant activity (one rotor) and rotor breakup and fibrillatory activity were studied. Our GPU implementation accommodates large grid size for both simulations. We tested grid sizes up to a maximum of 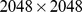. To ensure appropriate comparisons, the same model parameters were used for simulations running on CPUs and GPUs for each model. The CPU results were obtained from running on the machine that hosted the Fermi GPU.

For the single rotor simulations, we studied grid sizes ranging from 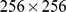 to 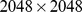. A total time of 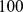 milliseconds was simulated and dt was varying from 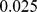 milliseconds to 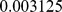 milliseconds, requiring from 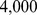 to 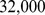 steps for simulations to finish. The number of nodes visited per second ranged from 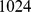 to 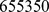. Square grids were used with an edge size of 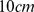.

**Figure 5:**
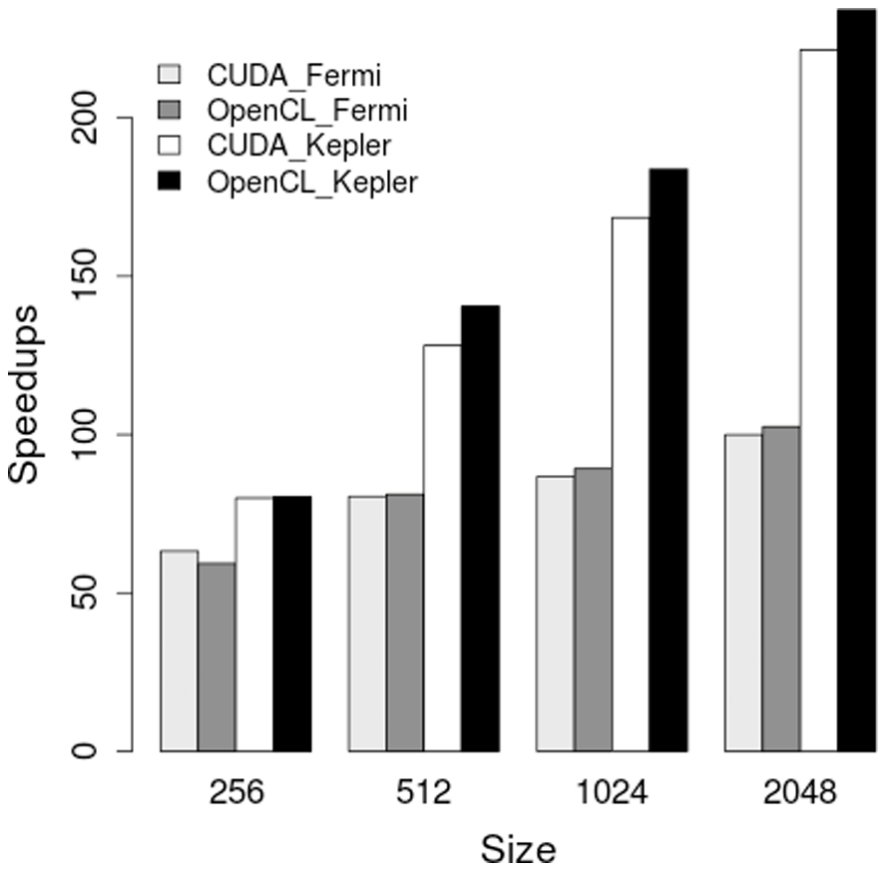
Speedups from running on the Fermi-architecture Tesla C2050 GPU and the Kepler architecture Tesla K20 GPU using OpenCL and CUDA for reentrant activity (one rotor) simulation.

Figure 5 shows the speedups provided by the Fermi GPU and the Kepler GPU over the sequential CPU implementation. In the figure, the X axis is the grid size from 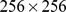 to 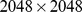, the Y axis is the relative speedup value, computed as CPU time/GPU time. The four bars represent the combinations of runs: OpenCL implementation running on two GPUs and CUDA implementation running on two GPUs.

For both CUDA and OpenCL GPU implementations, the Fermi GPU provided speedups of at least 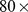 when running large grid sizes, such as 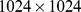, and 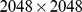; the Kepler GPU provided more than 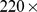 speedups for 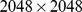 grid size. For each GPU card we observed that larger grid sizes provided larger speedups. Comparing the performance of the Fermi GPU with that of the Kepler GPU, we could see Kepler GPU was more than 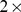 faster than Fermi GPU. The increase of the number of cores from 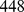 to 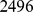 and the improvement of double-precision performance were among the factors contributing to the speedups. Since the Kepler results were obtained from running exactly the same code that ran on the non-Kepler GPUs, optimization might be applied specific to Kepler GPU for even more speedups. Although the GPU kernel function were equivalent for OpenCL implementation and CUDA implementation, OpenCL implementation was slightly faster than CUDA implementation on the two GPUs. One reason is that our OpenCL implementation predefined the total number of threads to be one while executing a kernel function that needed to be serialized; however, our CUDA implementation needed conditional statement to allow only one thread to run that kernel function which incurred overhead. Overall, both CUDA and OpenCL GPU implementations provided very good speedups on two different GPUs. It took the GPU implementations about 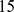 (or 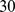) minutes to finish the largest simulated grid size (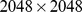) on Kepler GPU (or Fermi GPU) – the sequential CPU implementation took more than 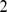 days. We noticed that although we copied back from GPU to CPU the transmembrane voltage values every constant timesteps (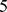 milliseconds of simulation), the IO overhead was very insignificant, especially for large grid size. So we excluded the IO time from the total running time for GPU implementations. The transmembrane voltage values for all nodes reside in GPU global memory. There is no need to copy the voltage values back to CPU in every single timestep. Fortunately, the GPUs we used contained enough global memory to hold the voltage values for each node even for the largest grid containing 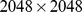 nodes.

Rotor breakup simulations were used to study performance during greater computational loads. In these tests we set the model parameters to simulate the breakup of a single rotor into multiple rotors, resulting in electrical activity that is much more complex than that of a single rotor (shown in Figure 2). The computational load for such simulations is typically higher because more nodes are active per unit time. Despite the fact that the rotor breakup simulation is much more complex, we observed similar significant speedups from GPUs.

### OpenACC Implementation

The hardware used to test OpenACC implementation is the machine hosting the Kepler GPU. To compile OpenACC code, we used HMPP 3.3.3 compiler from CAPS [27]. The compiler transforms the OpenACC code to either CUDA or OpenCL code as specified by the user.

**Figure 6:**
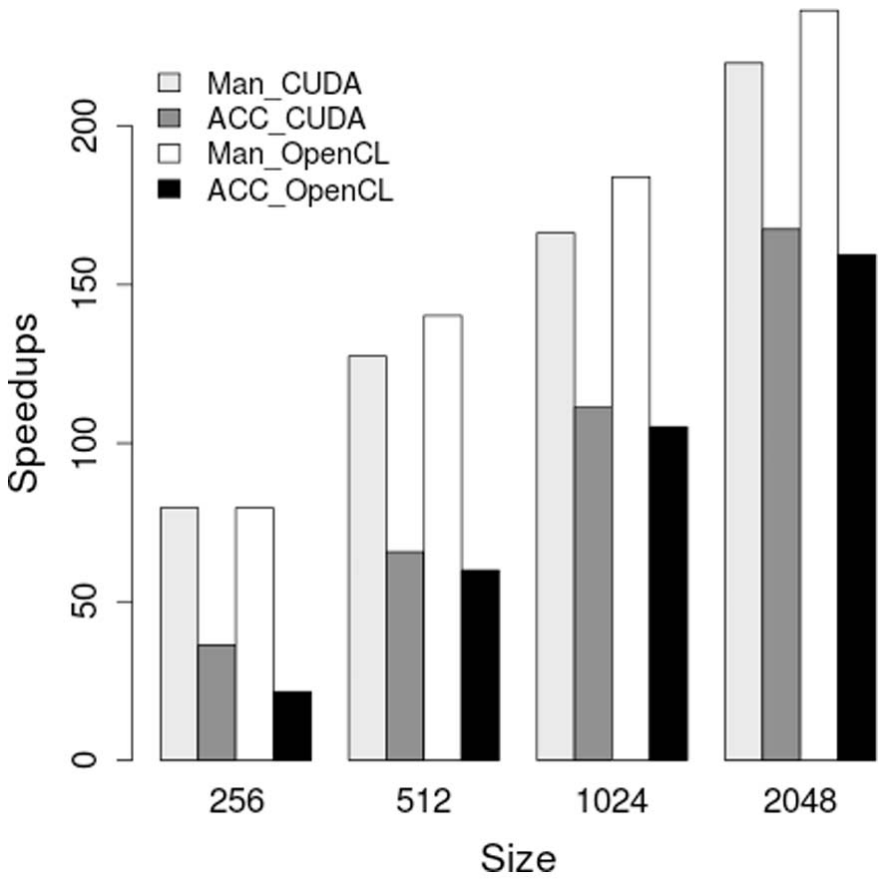
Speedups of hand-written GPU code (Man_CUDA, Man_OpenCL) over the sequential baseline vs. speedups of OpenACC targeting CUDA and OpenCL (ACC_CUDA, ACC_OpenCL) over the same baseline. All GPU codes were run on the Kepler GPU.

The comparison of speedups between automatically generated GPU code (CUDA/OpenCL) and hand-written GPU code (CUDA/OpenCL) is shown in Figure 6. The tests were run on Kepler GPU and the same reentrant activity as above was simulated. We can see from the figure that OpenACC code targeting CUDA and OpenCL achieved more than 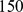 times speedup over sequential implementation with the largest input size and over 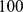 times speedup with the second largest input size. Although automatically generated GPU code did not achieve the same speedup as hand-written GPU code, the amount of modification to the original sequential code from OpenACC was trivial and much less than the hand-written counterpart. We believe OpenACC to be an efficient and effective way of exposing parallelism of potential applications.

### OpenMP Implementation

To provide more perspective for the speedups achieved by our GPU implementation, we conducted additional tests with a parallel CPU implementation of the model using OpenMP. The OpenMP implementation was run on the hardware platform that hosted Fermi GPU and with the same reentrant activity (one rotor) simulation input files. We achieved an average speedup of 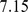 over sequential CPU implementation using 8 CPU threads.

**Figure 7:**
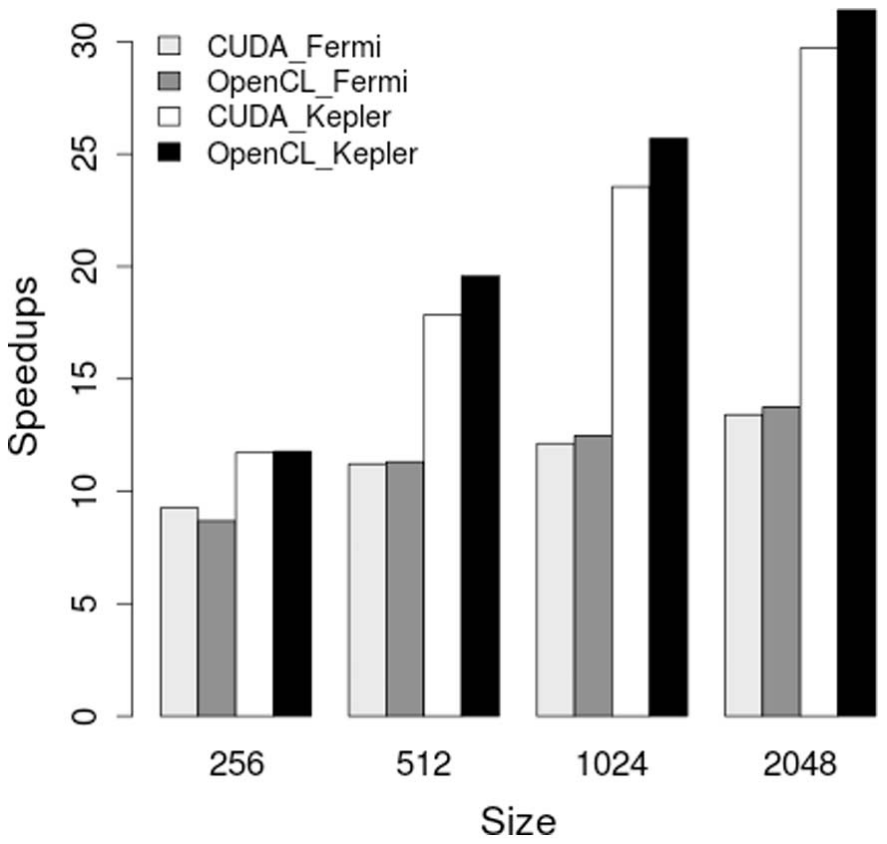
Speedups on Fermi GPU (NVIDIA C2050) and Kepler GPU (NVIDIA K20) over the 8 core OpenMP implementation. Reentrant activity (one rotor) was simulated.

Figure 7 shows the speedup results over OpenMP for hand-written CUDA and OpenCL implementations running on the Fermi GPU and the Kepler GPU. All GPU implementations provided more than 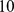 times speedup over the multi-core OpenMP implementation running over large input size on Fermi GPU. The speedup for both CUDA and OpenCL implementations increased quickly from about 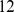 times to more than 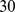 times running on Kepler GPU with the increase of problem size. The maximum speedup of 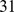 over OpenMP was achieved by running OpenCL implementation on the Kepler GPU.

### Implementations on MIC Architecture

We used Intel Xeon Phi 5110P coprocessor to test our hand-written OpenCL code, automatically generated OpenCL code (by OpenACC), and the OpenMP code written for Intel MIC architecture. The coprocessor contains 60 cores and supports 240 threads. The memory of the coprocessor is 8 GB. The machine hosting the coprocessor had 32 GB of memory and the CPU is Intel Xeon E5 clocked at 2.63 GHz. As to software, we used OpenCL 1.2 library (provided by Intel ICC compiler v14.0.0) for OpenACC and OpenCL implementations. The compiler to compile hand-written OpenCL code was GCC. We used the same CAPS compiler as above to compile OpenACC code to generate OpenCL code that runs on Intel MIC card. For OpenMP implementation, we used ICC compiler versioned at 14.0.0 from Intel.

**Figure 8:**
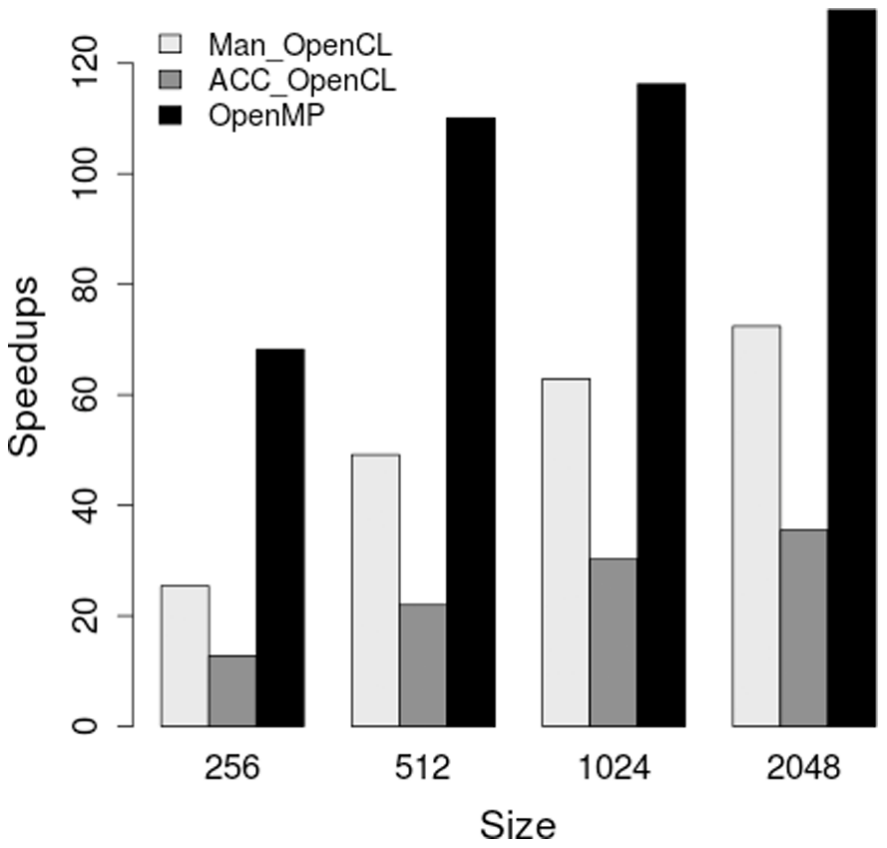
Speedups on MIC-architecture Xeon Phi coprocessor using hand-written OpenCL, OpenACC-generated OpenCL, and OpenMP implementation over the sequential implementation. Reentrant activity (one rotor) was simulated.

Figure 8 shows the speedups achieved on MIC accelerator from the three implementations. The OpenMP implementation was the fastest of the three. It achieved more than 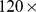 speedup for the largest simulation size. For smaller simulation size like 512 and 1024, it achieved more than 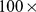 speedup. The MIC OpenMP implementation is vectorized with a factor of at least four because if we disable the vectorization, the speedup becomes four times less. The MIC OpenCL implementation remained identical to the implementation tested on GPUs, thanks to its portability. It provided decent speedups on MIC while offering such portability. For the largest simulation size, more than 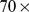 speedup was achieved. As the OpenCL library improves, the speedup number could get better. Different from the performance on GPUs, OpenACC only provided a maximum of 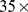 speedup on MIC. As it stands now, OpenMP is a better choice than OpenACC to get good performance on MIC.

## Discussion

In this section, we first summarize the optimizations applied to different implementations. Then we compare different programming languages with various metrics. In the end, we compare with the related work and summarize this work.

### Summary of Effective Optimizations for Different Implementations

Although it was straightforward to port the cardiac model to run on accelerators, optimizing the code was not trivial.

For CUDA and OpenCL implementations on GPU, we applied two other effective optimizations before optimizing the block size.

- Eliminating atomic operations. A direct port of the sequential code contained 9 equations that represent the updates (writes) from a current node to the 8 neighboring nodes and itself. Before optimization, atomic operations (in CUDA) and kernel isolations (in OpenCL) were used to avoid racing updates. Atomic operations are expensive and kernel isolations bring synchronization overhead. To address this limitation, we came up with a neighbor-update-free strategy. In this optimization strategy, each node “collects” (reads) update requests from the neighbors and performs the update to the node itself.
- Coalescing memory accesses. Coalesced memory accesses on GPUs means the data requests (e.g. by 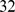 threads) consisting of contiguous (e.g. 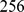 bytes for double) aligned memory space could be fulfilled by one memory access. More than one memory access will be needed otherwise. We achieved coalesced memory access by changing Array of Structures (AoS) to Structure of Arrays (SoA). Without coalesced memory access, the speedup numbers would get cut by at least a half.

We obtained the OpenACC implementation from adding pragmas to a sequential implementation that incorporated the above two optimizations. Added with the optimization for gang, worker, and vector configuration, we achieved good speedups from OpenACC on GPUs.

For MIC OpenMP implementation, as aforementioned, we applied loop unswitching optimization in addition to the above optimizations so that the vectorization power of MIC card could be better exploited. The loop unswitching strategy improved the speedup results by approximately 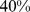.

For the sequential and OpenMP version that run on CPUs and serve as the baselines, we passed O3 optimization flag to the compiler so that the implementations were well optimized to be strong baselines.

### Parallel Programming Tools Comparison

In this section, we compare metrics like lines of source code change to the original implementation, portability, time taken to program, of the CUDA, OpenCL, OpenACC, and OpenMP implementations. Table 1 shows the detailed ratings.

**Table 1:** Comparison of multiple metrics between different parallel programming implementations for the cardiac wave propagation model.

Language	Code-Change	Estimated Time to Program	Platforms
CUDA	500(+/−)	Weeks	NVIDIA GPUs
OpenCL	500(+/−)	Weeks	GPUs, CPUs, MIC accelerator
OpenACC	10(+)	Days	GPUs, CPUs, MIC accelerator
OpenMP	10(+)	Days	CPUs, MIC accelerator

The Code-Change (second column) reports the estimated number of lines of code change for the kernel computation functions from the sequential C code. The “+” symbol after the number means addition and “+/−” means additions and deletions. There are about 220 lines of computation code in the sequential CPU implementation. The CUDA and OpenCL implementation needed to replace almost every statement of the original CPU implementation. Plus, they needed to include GPU initialization and data preparation code. The code change was mostly addition and replacement of statements. The OpenACC implementation only incurred about 10 statement additions to the sequential code. The initialization of GPU, data initialization for GPU, and data movement between GPU and CPU were all automatically handled in the generated CUDA and/or OpenCL code. The programmer only needed to figure out the correct pragmas and associated pragma attributes to add. The OpenMP implementation was also obtained by only placing a few pragmas around the parallel code regions. Considering the difficulty of programming using the languages (third column), CUDA and OpenCL are about the same, they could take an experienced programmer weeks to get the program correct and optimized for GPUs. In our case, it took us months to get the well-tuned CUDA version. In contrast, OpenACC and OpenMP program mostly involved figuring out where to place the pragmas and what parameters should go with these pragmas. It does not require the programmer to be as experienced as for CUDA programming. It took us less than a week to get the efficient OpenACC implementation and the baseline OpenMP implementation. We also compare the portability of these implementations (fourth column). The CUDA implementation can only run on NVIDIA GPUs while OpenCL can run across different architectures include GPUs, CPUs and accelerators like Intel Xeon Phi coprocessor. Since OpenACC implementation can be compiled to OpenCL code, it can target the same architectures as OpenCL can. The OpenMP program cannot run on GPUs, however, it can run on CPUs and Intel's Xeon Phi coprocessor (accelerator).

Combining the performance results and the above metrics, we can see the OpenCL implementation achieved the best speedups and portability on GPUs; the OpenACC implementation, taking the minimum amount of effort to program, also achieved very good speedups on GPUs and the same portability as OpenCL implementation did. For Intel MIC architecture, the OpenMP implementation was the performance champion. We attribute this partially to the performance differences between the compilers and the (OpenCL) libraries used to generate the executables.

### Related Work

The first published simulation of 2D wave propagation in cardiac arrhythmias model using GPU hardware was performed on an Xbox 360 and resulted in a speedup of more than 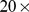 for specific model parameters [28]. Our work is the first to report the simulation results of the 2D wave propagation using CUDA, OpenCL, and OpenACC to manually ( CUDA/OpenCL) and automatically (OpenACC) take advantage of the power of modern computational accelerators like GPUs and Intel Xeon Phi coprocessor. Wienke et al. were the first to report the experiences with real-world applications using OpenACC [29]. Their OpenACC implementation achieved a fraction of 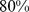 of the best performance offered by OpenCL implementation for one application and only 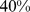 for a more complex medical program. As shown in Figure 6, our OpenACC implementation could achieve about 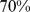 of the best hand-written CUDA/OpenCL implementation. In this work, we also showed that the OpenACC implementation outperformed a non-optimized hand-written OpenCL code on the Intel MIC accelerator (Figure 8). Hart et al. first showed that OpenACC could be used in massively-parallel, GPU-accelerated supercomputers [30].

Work that compared OpenACC, OpenCL, and CUDA include the acceleration of hydrocodes [31] and the acceleration of financial applications [15]. Our case study is focused on a different codebase (2D wave propagation). While the work of accelerating hydrocodes showed that OpenCL performed the worst, we reported that the OpenCL implementation could achieve both good performance and portability. Different from the work of accelerating financial applications, we went a further step to compare the performance of the OpenCL, OpenACC, and OpenMP implementation on Intel MIC accelerator. There are also a few work that focused on comparing the different implementations of the OpenACC directive-based language itself [32], [33]. Other than comparing the OpenACC related implementations, Oliveira et al. compared the CUDA, OpenCL, and OpenGL implementations of the cardiac monodomain equations [34]. In their work, the OpenCL implementation was slower than the CUDA implementation. Our work showed that both the hand-written OpenCL code and the OpenACC generated OpenCL code can perform as good as or even better than CUDA code while achieving portability.

### Summary

This paper builds on our previous report [35], by presenting our more efficient GPU implementations to dramatically improve the scalability of our model for GPU architecture. More importantly, we auto-parallelized the sequential code using OpenACC, the speedup of which was impressive–as much as 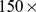 faster than the sequential version while the modification to the original sequential program was minimum. We have also compared the performance of parallel GPU computations with parallel CPU computations (using OpenMP). Our GPU implementation was as much as 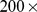 faster than the original sequential CPU code. The OpenMP code achieved 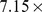 speedups over the sequential CPU code on average as displayed. Comparing with the OpenMP code, our GPU implementation reached more than 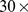 speedups on the Kepler GPU. We also tested with different implementations on Intel MIC architecture accelerator where the OpenMP implementation achieved the best speedup of more than 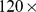 and the portable OpenCL implementation achieved more than 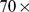 speedup.

We conclude that emerging software developments like the OpenACC directive-based programming language facilitate exploiting application parallelism offered by evolving hardware architecture. Our method of using OpenACC, OpenCL, and OpenMP to achieve efficient and effective parallelization on different accelerators can be generally applied to benefit other domains.

## Supporting Information

Software S1 Software Package containing all the implementations.
(ZIP)

## References

[1] Su X, Xu J, Ning K (2011) Parallel-META: A high-performance computational pipeline for metagenomic data analysis. In: 2011 IEEE International Conference on Systems Biology (ISB). pp. 173–178.

[2] Campbell D (2006) VSIPL++ acceleration using commodity graphics processors. In: HPCMP Users Group Conference, 2006. pp. 315–320.

[3] Spurzem R, Berczik P, Nitadori K, Marcus G, Kugel A, et al.. (2010) Astrophysical particle simulations with custom GPU clusters. In: 2010 IEEE 10th International Conference on Computer and Information Technology (CIT). pp. 1189–1195.

[4] Michalakes J, Vachharajani M (2008) GPU acceleration of numerical weather prediction. In: IEEE International Symposium on Parallel and Distributed Processing, 2008 (IPDPS 2008). pp. 1–7.

[5] Daga M, Feng W, Scogland T (2011) Towards accelerating molecular modeling via multi-scale approximation on a GPU. In: 2011 IEEE 1st International Conference on Computational Advances in Bio and Medical Sciences (ICCABS). pp. 75–80.

[6] MIC. Intel Many Integrated Core architecture. Available: http://www.intel.com/content/www/us/en/architecture-and-technology/many-integrated-core/intel-many-integrated-core-architecture.html. Accessed 2013 Dec 23.

[7] Neic A, Liebmann M, Hoetzl E, Mitchell L, Vigmond E, et al (2012) Accelerating cardiac bidomain simulations using graphics processing units. Biomedical Engineering, IEEE Transactions on 59: 2281–2290. 10.1109/TBME.2012.2202661 PMC3696513 22692867

[8] Mirin AA, Richards DF, Glosli JN, Draeger EW, Chan B, et al. (2012) Toward real-time modeling of human heart ventricles at cellular resolution: simulation of drug-induced arrhythmias. In: Proceedings of the International Conference on High Performance Computing, Networking, Storage and Analysis. Los Alamitos, CA, USA: IEEE Computer Society Press, SC' 12, pp. 2:1–2: :11.

[9] Richards DF, Glosli JN, Draeger EW, Mirin AA, Chan B, et al (2013) Towards real-time simulation of cardiac electrophysiology in a human heart at high resolution. Computer Methods in Biomechanics and Biomedical Engineering 16: 802–805. 23734785 10.1080/10255842.2013.795556

[10] Niederer S, Mitchell L, Smith N, Plank G (2011) Simulating human cardiac electrophysiology on clinical time-scales. Frontiers in Physiology 2. 10.3389/fphys.2011.00014 PMC3079856 21516246

[11] Pope B, Fitch B, Pitman M, Rice J, Reumann M (2011) Performance of hybrid programming models for multiscale cardiac simulations: Preparing for petascale computation. Biomedical Engineering, IEEE Transactions on 58: 2965–2969. 10.1109/TBME.2011.2161580 21768044

[12] Pope BJ, Fitch BG, Pitman MC, Rice JJ, Reumann M (2011) Petascale computation performance of lightweight multiscale cardiac models using hybrid programming models. In: Engineering in Medicine and Biology Society,EMBC, 2011 Annual International Conference of the IEEE. pp. 433–436. 10.1109/IEMBS.2011.6090058 22254341

[13] CUDA. What is CUDA. Available: http://developer.nvidia.com/what-cuda. Accessed 2013 Dec 23.

[14] OpenCL. OpenCL–the open standard for parallel programming of heterogeneous systems.Available: http://www.khronos.org/opencl. Accessed 2013 Dec 23.

[15] Grauer-Gray S, Killian W, Searles R, Cavazos J (2013) Accelerating financial applications on the GPU. In: Proceedings of the 6th Workshop on General Purpose Processor Using Graphics Processing Units. New York, NY, USA: ACM, GPGPU-6, pp. 127–136.

[16] OpenACC. OpenACC–directives for accelerators. Available: http://www.openacc-standard.org. Accessed 2013 Dec 23.

[17] ten Tusscher K, Panfilov A (2005) Wave propagation in excitable media with randomly distributed obstacles. Multiscale Modeling & Simulation 3: 265–282.

[18] Su L, Wang W, Wang H (2011) A characteristic difference method for the transient fractional convection-diffusion equations. Appl Numer Math 61: 946–960.

[19] Zhang Z, Wang Y, Wang Q (2011) A characteristic centred finite difference method for a 2D air pollution model. Int J Comput Math 88: 2178–2198.

[20] Tessmer E (2000) Seismic finite-difference modeling with spatially varying time steps. GEOPHYSICS 65: 1290–1293.

[21] Welter M, Rieger H (2013) Interstitial fluid flow and drug delivery in vascularized tumors: A computational model. PLoS ONE 8: e70395. 23940570 10.1371/journal.pone.0070395 PMC3734291

[22] Agladze K, Kay MW, Krinsky V, Sarvazyan N (2007) Interaction between spiral and paced waves in cardiac tissue. American Journal of Physiology - Heart and Circulatory Physiology 293: H503–H513. 17384124 10.1152/ajpheart.01060.2006 PMC3019092

[23] Kay MW, Gray RA (2005) Measuring curvature and velocity vector fields for waves of cardiac excitation in 2-D media. Biomedical Engineering, IEEE Transactions on 52: 50–63. 10.1109/TBME.2004.839798 15651564

[24] Drouhard JP, Roberge FA (1987) Revised formulation of the Hodgkin-Huxley representation of the sodium current in cardiac cells. Computers and Biomedical Research 20: 333–350. 3621918 10.1016/0010-4809(87)90048-6

[25] Beeler GW, Reuter H (1977) Reconstruction of the action potential of ventricular myocardial fibres. The Journal of Physiology 268: 177–210. 874889 10.1113/jphysiol.1977.sp011853 PMC1283659

[26] OpenMP. OpenMP–the OpenMP API specification for parallel programming. Available: http://openmp.org/wp/. Accessed 2013 Dec 23.

[27] CAPS. CAPS compilers. Available: http://www.caps-entreprise.com/products/caps-compilers/. Accessed 2013 Dec 23.

[28] Scarle S (2009) Implications of the Turing completeness of reaction-diffusion models, informed by GPGPU simulations on an XBox 360: Cardiac arrhythmias, re-entry and the Halting problem. Computational Biology and Chemistry 33: 253–260. 19577519 10.1016/j.compbiolchem.2009.05.001

[29] Wienke S, Springer P, Terboven C, an Mey D (2012) OpenACC: first experiences with real-world applications. In: Proceedings of the 18th international conference on Parallel Processing. Berlin, Heidelberg: Springer-Verlag, Euro-Par' 12, pp. 859–870.

[30] Hart A, Ansaloni R, Gray A (2012) Porting and scaling OpenACC applications on massivelyparallel, GPU-accelerated supercomputers. European Physical Journal - Special Topics 210: 5–16.

[31] Herdman JA, Gaudin WP, McIntosh-Smith S, Boulton M, Beckingsale DA, et al.. (2012) Accelerating hydrocodes with OpenACC, OpeCL and CUDA. In: Proceedings of the 2012 SC Companion: High Performance Computing, Networking Storage and Analysis. Washington, DCUSA: IEEE Computer Society, SCC' 12, pp. 465–471.

[32] Reyes R, López I, Fumero JJ, Sande F (2013) A preliminary evaluation of OpenACC implementations. J Supercomput 65: 1063–1075.

[33] Reyes R, Lopez I, Fumero J, De Sande F (2012) Directive-based programming for GPUs: A comparative study. In: High Performance Computing and Communication 2012 IEEE 9th International Conference on Embedded Software and Systems (HPCC-ICESS). pp. 410–417.

[34] Oliveira RS, Rocha BM, Amorim RM, Campos FO, Meira W, et al.. (2012) Comparing CUDA, OpenCL and OpenGL implementations of the cardiac monodomain equations. In: Proceedings of the 9th international conference on Parallel Processing and Applied Mathematics - Volume Part II. Berlin, Heidelberg: Springer-Verlag, PPAM' 11, pp. 111–120.

[35] Wang W, Huang HH, Kay M, Cavazos J (2011) GPGPU accelerated cardiac arrhythmia simulations. In: Engineering in Medicine and Biology Society, EMBC, 2011 Annual International Conference of the IEEE. pp. 724–727. 10.1109/IEMBS.2011.6090164 PMC3589987 22254412

